# Pharmacological Effects of “Jutsu” (*Atractylodis rhizome* and *Atractylodis lanceae rhizome*) on 1-(2,5-Dimethoxy-4-iodophenyl)-2-aminopropane (DOI)-Induced Head Twitch Response in Mice (I)

**DOI:** 10.3390/molecules190914979

**Published:** 2014-09-18

**Authors:** Chiaki Murayama, Ching-Chiung Wang, Seiwa Michihara, Hisayoshi Norimoto

**Affiliations:** 1 Kampo Research Laboratories, Kracie Pharma Ltd., Kanebo machi 3-1, Takaoka, Toyama 933-0856, Japan; E-Mails: murayama_chiaki@phm.kracie.co.jp (C.M.); michihara_seiwa@phm.kracie.co.jp (S.M.); 2 School of Pharmacy, College of Pharmacy, Taipei Medical University, 250 Wu-Hsing Street, Taipei 110, Taiwan; E-Mail: crystal@tmu.edu.tw

**Keywords:** hallucinations, *Atractylodis rhizome* (Byaku-jutsu), *Atractylodis lanceaerhizome* (So-jutsu), atractylenolide III, β-eudesmol, DOI-induced head-twitch

## Abstract

Hallucinations are a common non-motor symptom of Parkinson’s disease and various forms of dementias. Yokukansan and Yokukansankachimpihange have attracted attention due to their effectiveness in the treatment of hallucinations of dementia. To clarify which component in these formulas contribute to the effects, at first, we focused on their differences in compositions to examine the pharmacological effects on the selective 5-HT_2A/2C_ agonist 1-(2,5-dimethoxy-4-iodophenyl)-2-aminopropane (DOI)-induced head-twitch response (HTR) in mice that has been used as animal hallucination model. Results indicated that water extract of Byaku-jutsu (*Atractylodes japonica*) showed a stronger inhibitory effect on DOI-induced HTR than that of So-jutsu (*A. lancea*) corresponding to their major constituents of atractylenolide III and β-eudesmol, and suggested that the major constituents should be active constituents contributing to the antihallucination effects of Byaku- and So-jutsu. Besides, the part B–C ring (butenolide) in atractylenolide III was found to be similar to the structure of serotonin and suggested that the B–C ring may partially play role in antagonistic activity against serotonin receptors. Thus, a novel, rational design of butenolide-related compounds may as potential lead compounds for new drug development. Analysis of the chemical components of Byaku- and So-jutsu and further study on their structure-activity relationships are currently in progress.

## 1. Introduction

Hallucinations are a common non-motor symptom of Parkinson’s disease and various forms of dementias such as Alzheimer’s disease and the lewy body dementias [1]. Besides, hallucinations are known for occurring due to the consumption of psychoactive substances such as deliriants and psychedelics. Recently, smoking synthetic cannabis, known as “dappo (law-evading) herb” in Japan, has become a big social problem in that some smokers have caused fatal traffic accidents due to hallucinations. However, an effective drug therapy for hallucinations has yet not been established, although benzodiazepines for sedation and/or overdose of the antimuscarinic agents with infusions of physostigmine can be used [2].

On the other hand, in recent years, the traditional Chinese-Japanese medicines (also Kampo medicines), Yokukansan [3,4] and Yokukansankachimpihange (YKH) [5,6] have been demonstrated to be effective in the treatment of hallucinations of dementia patients by clinical studies and have attracted great attention in Japan. In addition, Yokukansan has been reported to inhibit the selective 5-HT_2A/2C_ agonist 1-(2,5-dimethoxy-4-iodophenyl)-2-aminopropane (DOI)-induced head-twitch response (HTR) in mice, that has been used as animal hallucination model [7,8].

In our previous study, we also found that YKH not only reduced DOI-induced HTR in normal mice, but also reduced DOI-induced HTR in senescence-accelerated mice [9], and suggesting that YKH may be superior to Yokukansan in its effects on hallucination. Yokukansan consists of Atractylodis or Atractylodis lanceae rhizoma, Poria, Cnidii rhizoma, Radix Angelicae, Radix Bupleuri, Radix Glycyrrhizae, and Uncaria hook, while YKH is a combination of Yokukansan with Citrus unshiu peel and Pinellia tuber.

Therefore, in the order to clarify which component(s) in these formulas contribute to the pharmacological action against hallucination, we at first focused on the differences in composition between Yokukansan and YKH, namely, *Atractylodis* or *Atractylodis lanceae rhizome*, *Citrus unshiu* peel and *Pinellia tuber*, for further pharmacological study. As a result, we found that “Jutsu” (*Atractylodis rhizome* and *Atractylodis lanceae rhizome*) showed antihallucination-like effects in DOI-induced HTR tests.

In the Japanese pharmacopoeia “Jutsu” is traditionally divided into “Byaku-jutsu” (*Atractylodis rhizome*) and “So-jutsu” (*Atractylodis lanceae rhizome*), which are defined as the rhizome of *Atractylodes japonica* Koidzumi ex Kitamura, or the rhizome of *A. ovata* De Candolle and *A. lancea* De Candolle, and *A. chinesis* Koidzumi or their interspecific hybrids, according to their different applications [10]. The former as a *qi* (energy)-invigorator, and the latter as an aromatic dampness-transforming medicine, and are usually used for treatment of psychological, immunologic and digestive disorders. Besides, chemical differences have been found, whereby atractylon is the major constituent in Byaku-jutsu, and β-eudesmol and hinesol are the major coomponents in So-jutsu, respectively [11].

In this study, we report the pharmacological effects of water extracts of Byaku-jutsu (*A. japonica* Koidzumi ex Kitamura) and So-jutsu (*A. lancea* De Candolle) on DOI-induced HTR in normal mice. In the meantime, atractylenolide III and β-eudesmol were targeted firstly to know whether these constituents contribute to the pharmacological effects, because atractylon is an excessively unstable sesquiterpenoid in Byaku-jutsu, which is easily converted into atractylenolide II, and atractylenolide III in particular [11,12].

## 2. Results and Discussion

### 2.1. Effects of Byaku-Jutsu and So-jutsu Extracts on DOI-Induced HTR in Mice

**Figure 1:**
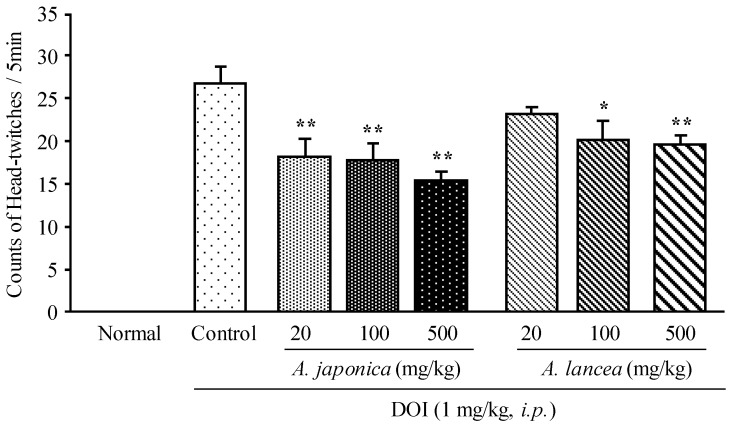
Effect of Byaku-jutsu and So-jutsu extracts on DOI-induced HTR in mice. Each column represents the mean ± S.E. of nine mice. * *p* < 0.05, ** *p* < 0.01 *vs.* Control (one-way ANOVA followed by Dunnett’s test).

Consistent with a published report [13], kantaserin tartrate (0.25 mg/kg, *i.p.*), a highly selective antagonist for 5-HT_2A_ receptors served as a positive control, and was shown to significantly decrease the DOI-induced HTR (data not shown) in our preliminary test, indicating that the hallucination model of mice was successfully established. As shown in Figure 1, DOI induced markedly HTR in control, while no HTR were observed in normal animals. The effects of consecutive administration of various doses of Byaku-jutsu and So-jutsu (both at 20, 100, 500 mg/kg) on DOI-induced HTR were evaluated, and Byaku-jutsu significantly decreased the DOI-induced HTR in a dose-dependent manner. Similarly, So-jutsu dose-dependently decreased the DOI-induced HTR, with particularly significant effects at doses of 100 and 500 mg/kg.

In addition, Byaku-jutsu showed stronger effects on the DOI-induced HTR than those of So-jutsu although no significant differences between them were seen at each dose, respectively.

### 2.2. Effects of Atractylenolide III and β-Eudesmol on DOI-Induced HTR in Mice

**Figure 2:**
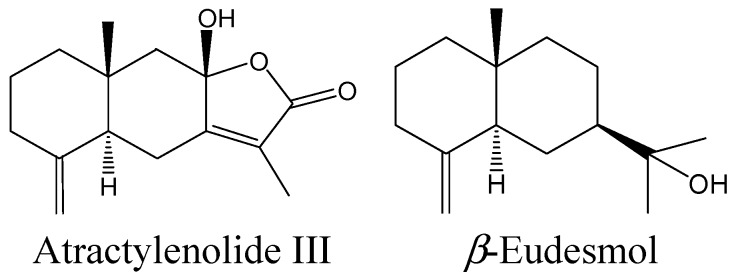
Chemical structures of atractylenolide III and β-eudesmol.

As described above, both Byaku-jutsu and So-jutsu are characterized by the presence of abundant essential oils, and among their chemical differences atractylon was found to be the major constituent in Byaku-jutsu, and β-eudesmol and hinesol were the most abundant components in So-jutsu, respectively [11]. Furthermore, atractylon is easily oxidized into atractylenolides II and III at room temperature [11,12]. Thus, in this study, atractylenolide III and β-eudesmol (Figure 2) were further used to clarify whether they were the active constituents in each extract.

**Figure 3:**
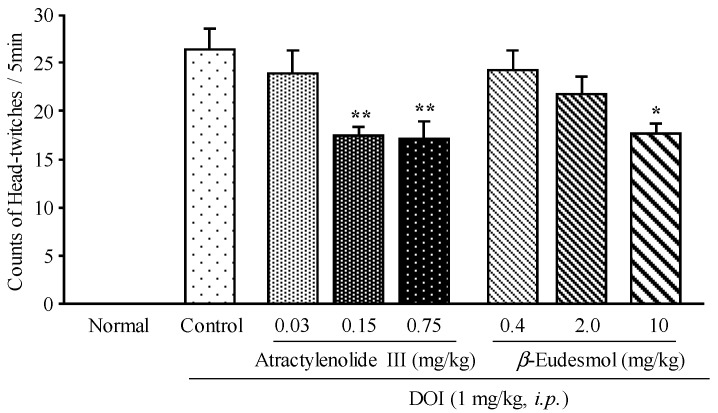
Effect of atractylenolide III and β-eudesmol on DOI-induced HTR in mice. Each column represents the mean ± S.E. of 9 mice. * *p* < 0.05, ** *p* < 0.01 *vs.* Control (one-way ANOVA followed by Dunnett’s test).

On the basis of the results of our previous chemical study (indicating on average a 0.15% of actractylenolide III and 2.0% of β-eudesmol contained in each extract), various doses of atractylenolide III (0.03, 0.15, or 0.75 mg/kg) or β-eudesmol (0.4, 2.0, 10 mg/kg) were used to evaluate their effects on DOI-induced HTR. Both compounds dose-dependently inhibited the DOI-induced HTR compared to the control (Figure 3), and the effects were parallel to the results observed with Byaku-jutsu and So-jutsu extracts. This result suggested that atractylenolide III and β-eudesmol could contribute to the inhibitory activity against DOI-induced HTR of Byaku-jutsu and So-jutsu extracts, respectively.

However, atractylenolide III also was detected in So-jutsu extract although its amount was approximately 50 times less than that in Byaku-jutsu extract. Furthermore, both compounds that seem to contribute to the effects on DOI-induced HTR share a similar structure with a eudesmane skeleton. Besides atractylenolide III or β-eudesmol, Byaku-jutsu and/or So-jutsu contain other eudesmane-type sesqiterpenoids such as atractylon, atractylenolide I and II, and β-selinene, thus further chemical study on their isolation and purification is advised, rather than limiting the chemical study of the water extract. Interestingly, biatractylolide, a dimer of atractylenolide III, was reported to improve the studying memory of the Alzheimer disease’s rat models induced by Aβ_1-40_ [14], and by AlCl_3_ [15]. Head-twitches in mice, induced by DOI, are thought to be mediated via central 5-HT_2A_ receptors [16], therefore, as described above, ketanserin, a highly selective antagonist for 5-HT_2A_ receptors, was also examined in our pre-test and observed to decrease significantly the DOI-induced HTR. In addition, both Byaku-jutsu and So-jutsu did not show any effects on locomotor activity of the mice (data not shown) and the results are in consistent with a report from Kobayashi [17]. Therefore, we speculated that Byaku-jutsu, So-jutsu and their active constituents may act through the serotonin pathway to show their antihallucination-like effects.

**Figure 4:**
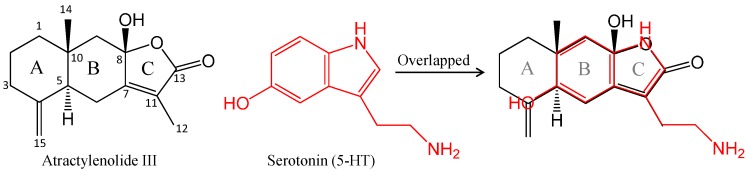
Postulated active site in the chemical structure in atractylenolide III compared to serotonin (5-HT).

Note worthily, atractylenolide III shows a very similar (butenolide or hydroxybutenolide) B–C ring structure to serotonin (5-HT) (Figure 4), and the B–C ring part also has similarity to some extent with the quinazoline-2,4-dione part of ketanserin, although the stereostructure of the B–C ring is not as flat as serotonin. 

Moreover, atractylenolide III showed a stronger inhibitory effect on the DOI-induced HTR than that of β-eudesmol. Interestingly, it has been reported that 6-fluro-N,N-diethyltryptamine, a mimic of serotonin, lacked DOI-like activity in spite of having a comparable 5-HT receptor affinity [18], and suggesting that an aromatic structure may not be the determinant site in the hallucinatory or antihallucinatory effects. On the other hand, most known anti-hallucinogens such as ketanserin have various side effects due to a complex interaction of serotonin, adrenergic, and histamine receptor systems which all share a similar aromatic-structure. In other words, it is a B–C ring flexible structure that may account for the ability of atractylenolide III to interact with the 5-HT receptors but distinctly from the known antagonists. In fact, the body weights of mice were recorded every day during administration and no differences among groups were observed. Taken together, it suggested that the B–C ring part may partially play a role in the antagonistic activity against 5-HT receptors.

## 3. Experimental Section

### 3.1. Experimental Animals

Male ICR mice (four weeks old, 17–19 g on arrival) were purchased from SLC Inc. (Shizuoka, Japan). The animals were reared in an air-conditioned animal house facility (room temperature 23 ± 2 °C; 12 h light/dark cycle; relative humidity 55% ± 10%) at Kampo Research Laboratories in Kracie Pharma Ltd (Takaoka, Japan). The mice were housed in sterilized polypropylene cages (3 mice/cage) and provided laboratory pellet chow (CE-2, Clea Japan Inc., Tokyo, Japan) and water *ad libitum*. Before experimental procedures, they were acclimated to the room for one week. This experiment (approval No.100240, 100259) was reviewed and approved by Experimental Animal Care Committee of Kracie Pharma Ltd. (Takaoka, Japan).

### 3.2. Chemicals and Extract Preparation of Byaku-Jutsu and So-Jutsu

DOI (Lot No.012M4812V) was purchased from Sigma-Aldrich (St. Louis, MO, USA). Ketanserin Tartrate (Lot No. CDL1847) was purchased from Wako Pure Chemical Industries Ltd (Tokyo, Japan). They were dissolved in saline and were injected intraperitoneally in a volume of 1 mL/kg. Control animals were injected with saline.

The rhizomes of *A. japonica* Koidzumi ex Kitamura (Lot No. 1106C009302, collected from Heilongjiang Province, P.R. China) were purchased from Tochimoto Tenkaido Co., Ltd. (Tokyo, Japan), and *A. lancea* (Lot No. OFS22K06, collected from Hubei Province, China) was purchased from Kanai Tokichi Shoten Co., Ltd. (Tokyo, Japan), and they were identified by the R&D Center Laboratory, Sheng Chang Pharmaceutical Co., Ltd. (Taipei, Taiwan) with certifications No. SC01120507 and No. SC01120505, respectively. In addition, their voucher specimens are deposited at the Herbarium of Kampo Research Laboratories, Kracie Pharma, Ltd. (Takaoka, Japan).

The dried rhizomes of *A. japonica* and *A. lancea* (50 g and 1.0 kg) was pulverized and extracted with 0.2 or 10 L water under reflux for 30 min, and then lyophilized to give extract with yields of 23.4% and 30.6%, respectively. The powdered extract was suspended in distilled water just before use and administered to experimental animals by gastric administration at a volume of 10 mL/kg.

Atractylenolide III (Lot No. WEN2356) and β-eudesmol (Lot No.WEG2032) were purchased from Wako Pure Chemical Industries Ltd. (Tokyo, Japan) and were dissolved in corn oil (Lot No.2012H2137, Junsei Chemical Co., Ltd., Tokyo, Japan), while control animals were orally administered with corn oil at a volume of 3.3 mL/kg.

### 3.3. DOI-Induced Head Twitch Response Experiments

DOI-induced HTR experiment was carried out according to the method described by Darmani *et al*. [13]. Briefly, mice were place in a quiet testing room for 1 h before DOI injection. After mice were injected with saline or 1 mg/kg DOI, each mouse was placed in a separated plastic breeding cage (172 × 240 × 129 mm) for 5 min and then the numbers of HTR were recorded by a video camera and/or counted by an observer with 5 min. The head twitch response is a typical of paroxysmal head-twitching behavior that is easily distinguished from head bobbing, lateral movements of the head from side to side, or grooming.

Before yhe DOI-induced HTR experiment, mice were randomly assigned to various treatment groups (nine mice/group), and were consecutively given Byaku-jutsu and So-jutsu extracts, and their main chemical constituents atractylenolide III and β-eudesmol for 13 days, respectively. The mice were allowed to habituate to the testing room for 1 h after the last administration, and the subjected to the DOI injection and the measurement of HTR. In addition, body weights were recorded every 1 day.

### 3.4. Statistical Analysis

The data are expressed as mean ± standard error of means (S.E.M). Significant differences were determined by one-way analysis of variance (ANOVA) followed by Dunnett’s test for multiple comparisons. *p* values of less than 0.05 were considered statistically significant.

## 4. Conclusions

In the course of a study to clarify which components contribute to the effects on hallucination induced by DOI in mice between two Kampo formulas, Yokukansan and YKH, we found that “Jutsu” (*Atractylodis rhizome* and *Atractylodis lanceae rhizome*) showed antihallucination-like effects. Results indicated that water extracts of Byaku-jutsu (*A. japonica* Koidzumi ex Kitamura) showed a stronger inhibitory effect on DOI-induced HTR than that of So-jutsu (*A. lancea* De Candolle), although no significant difference were observed between two. Furthermore, to know whether the major constituents contribute to their pharmacological effects, atractylenolide III and β-eudesmol were evaluated and it was found that both showed significant effects against DOI-induced HTR. The results suggest that these major constituents could contribute to the pharmacological effects in each extract.

In addition, the effects of atractylenolide III on the DOI-induced HTR was stronger than that of β-eudesmol. When a comparison of their chemical structures between two constituents and serotonin or its 5-HT_2A_ receptor antagonist ketanserin was made, the B–C ring part (butenolide or hydroxy-butenolide) in atractylenolide III was found to be very similar to the structure of serotonin. These findings suggest that the B–C ring part may at least partially play a role in the antagonistic activity against 5-HT receptors. Thus, a novel, rational design of butenolide-related compounds may produce potential lead compounds for new drug development against hallucination.

At present, detailed chemical studies on the isolation and purification of components such as atractylon and actractylenolide I are underway, and further studies on their structure-activity relationship will reported in a follow-up paper. In addition, in order to further clarify the 5-HT receptor affinity and the pharmacokinetics and metabolic mechanism in the brain, both a receptor binding assay and a pharmacokinetic study by a combined liquid chromatography/NMR spectrometry/mass spectrometry (LC/NMR/MS) method are planned to be performed.
